# Immunoinformatic-based design of immune-boosting multiepitope subunit vaccines against monkeypox virus and validation through molecular dynamics and immune simulation

**DOI:** 10.3389/fimmu.2022.1042997

**Published:** 2022-10-13

**Authors:** Muhammad Suleman, Farooq Rashid, Shahid Ali, Hassan Sher, Sisi Luo, Liji Xie, Zhixun Xie

**Affiliations:** ^1^ Center for Biotechnology and Microbiology, University of Swat, Swat, Pakistan; ^2^ Division of Infectious Diseases, Chongqing Public Health Medical Center, Chongqing, China; ^3^ Centre for Plant Science and Biodiversity, University of Swat, Swat, Pakistan; ^4^ Department of Biotechnology, Guangxi Veterinary Research Institute, Nanning, China; ^5^ Guangxi Key Laboratory of Veterinary Biotechnology, Nanning, China; ^6^ Key Laboratory of China (Guangxi)-ASEAN Cross-border Animal Disease Prevention and Control, Ministry of Agriculture and Rural Affairs of China, Nanning, China

**Keywords:** monkeypox virus, immunoinformatics, vaccine design, molecular docking, MD simulation

## Abstract

Monkeypox virus is the causative agent of monkeypox disease, belonging to an orthopoxvirus genus, with a disease pattern similar to that of smallpox. The number of monkeypox cases have robustly increased recently in several countries around the world, potentially causing an international threat. Therefore, serious measures are indispensable to be taken to mitigate the spread of the disease and hence, under these circumstances, vaccination is the best choice to neutralize the monkeypox virus. In the current study, we used immunoinformatic approaches to target the L1R, B5R, and A33R proteins of the monkeypox virus to screen for immunogenic cytotoxic T-lymphocyte (CTL), helper T-lymphocyte (HTL), and B-cell epitopes to construct multiepitope subunit vaccines. Various online tools predicted the best epitope from immunogenic targets (L1R, B5R, and A33R) of monkeypox virus. The predicted epitopes were joined together by different linkers and subjected to 3D structure prediction. Molecular dynamics simulation analysis confirmed the proper folding of the modeled proteins. The strong binding of the constructed vaccines with human TLR-2 was verified by the molecular docking and determination of dissociation constant values. The GC content and codon adaptation index (CAI) values confirmed the high expression of the constructed vaccines in the pET-28a (+) expression vector. The immune response simulation data delineated that the injected vaccines robustly activated the immune system, triggering the production of high titers of IgG and IgM antibodies. In conclusion, this study provided a solid base of concept to develop dynamic and effective vaccines that contain several monkeypox virus-derived highly antigenic and nonallergenic peptides to control the current pandemic of monkeypox virus.

## Introduction

Monkeypox virus, a double-stranded DNA virus with a genome size of 197 kb, contain more than 197 nonoverlapping open reading frames (ORFs) (1, 2). Moreover, the virus also contains membrane proteins, structural proteins, and DNA-dependent RNA polymerase (3). The virus belongs to the family Poxviridae, subfamily Chordopoxvirinae, and genus Orthopoxvirus and is the causative agent of a rare zoonotic disease, monkeypox. Monkeypox is similar to smallpox; however, the signs and symptoms are less severe than those of smallpox (2).

The transmission patterns and infection routes of the monkeypox virus are undefined (1). However, it has been found that the current outbreak of the disease is due to sexual transmission of the virus among men; nevertheless, this virus could also disseminate through body fluids, scabs, and shared bedding (2).

Monkeypox virus was discovered in monkeys in 1958 at a Danish laboratory, while the first case in humans was reported in 1970 in the Democratic Republic of Congo (DRC), previously Zaire, in a nine months baby (4, 5). The virus was not reported outside Africa until 2003, but since then, it has spread into central and western African countries (5). Phylogenetic analyses have revealed that the virus has circulated undetected outside central and western Africa for some time (6). From 2018 to 2021, monkeypox cases were reported in the United States (US), United Kingdom (UK), Singapore, and Israel (7). Since May 2022, the virus has spread to more than 50 countries of the world, and the World Health Organization (WHO) on June 23, 2022, declared monkeypox an ‘evolving threat of modern public health concern’ (6).

Currently, no specific treatments are being devised to treat monkeypox. However, antivirals, vaccinia immune globulin (VIG), and smallpox vaccines are useful to control monkeypox outbreaks. As vaccines are limited, the WHO, on June 14, 2022, issued an interim guidance that mass vaccination is not needed for this disease (8). Due to the current pandemic situation of monkeypox, the development of vaccines against monkeypox virus is urgently needed. Statistical data showed that vaccination against smallpox with the vaccinia virus was 85% effective against monkeypox (9). It has been demonstrated that the L1R (intracellular mature virus specific) protein and B5R and A33R (extracellular enveloped specific) proteins are immunogenic in nature and conferred protection against the vaccinia virus (10). In particular, these proteins are necessary for the virus to bind to, fuse with, and enter target cells (11, 12). The L1R and B5R immunogens are the target of IMV and EEV neutralizing antibodies while the A33R is the target of complement mediated cytolysis (13, 14). Moreover, subunit recombinant vaccines of vaccinia virus using LIR, B5R, and A33R have also been shown to be effective against monkeypox (15).

Both cellular and humoral immunity are important for all orthopoxviruses including the monkeypox virus. However, cytotoxic T-cell are of great interest as they provide a better protection. CD8^+^ T cells protect against the disease, but CD4^+^ T cell dependent antibody production is indispensable in clearing the virus after acute infection. Moreover, CD4^+^ cells are more potent in the secretion of IFN-γ compared to CD4^+^ T cells (16, 17). Vaccination is one of the best choices to trigger immune responses and neutralize invading pathogens. It has been reported that vaccination is the most reliable and effective strategy against infectious diseases and prevents approximately 2-3 million deaths per year (WHO, 2007). During the recent pandemic of severe acute respiratory syndrome coronavirus 2 (SARS-CoV-2), immunoinformatics based subunit vaccines engineered with adjuvants and conformational linkers against B-cell epitope, helper T-lymphocytes (HTL) and cytotoxic T-lymphocyte (CTL) have been designed (18). Similarly, in another immunoinformatics study using spike protein of SARS-CoV-2, highly efficient, immunodominant CTL epitopes were predicted that generated specific and robust immune response (19). Therefore, in the present study, we used an immunoinformatic approach to target the L1R, B5R, and A33R proteins of the monkeypox virus to screen for immunogenic CTL, HTL, and B-cell epitopes to construct multiepitope subunit vaccines (MESVs) against monkeypox virus. The immunoinformatic approach has several useful characteristics over traditional vaccine development; for example, computationally designed vaccines are thermodynamically stable, more effective, low cost, highly specific, and less time consuming to develop. Furthermore, the constructed vaccine was modeled and checked for proper folding and stability through molecular dynamics (MD) simulations. Subsequently, the binding affinity of the designed vaccines with human toll like receptor 2 (TLR-2) was checked by a molecular docking approach. This study will provide cost-effective, highly immunogenic, and nonallergenic MESVs against the monkeypox virus to control the current pandemic.

## Materials and methods

### Sequence retrieval and immunogenic peptide prediction

**Figure 1:**
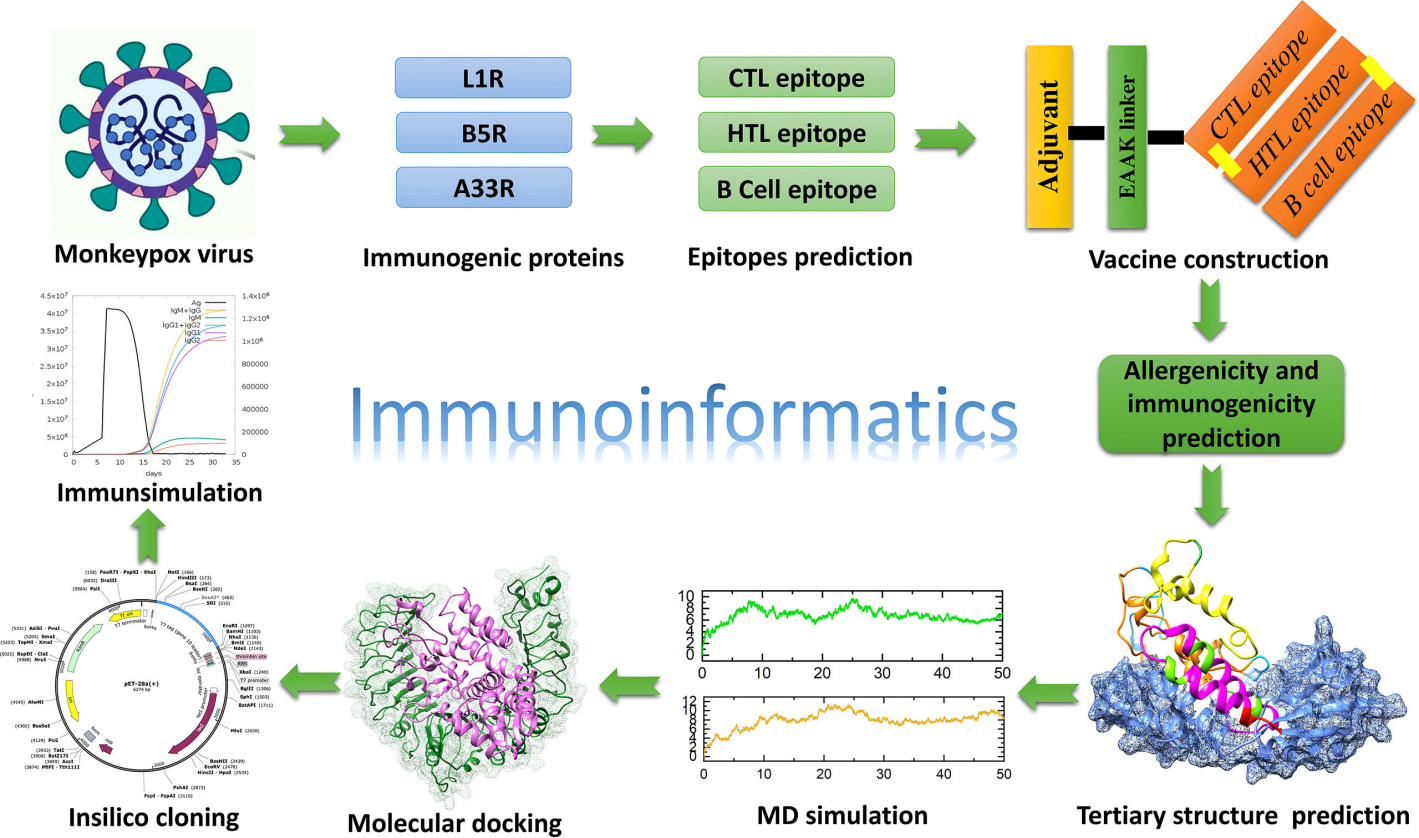
Overall workflow of the development of a multiepitope subunit vaccine targeting monkeypox virus using the immunoinformatic approach.

The protein sequences of the L1R (Accession ID: Q3I7N2), B5R (Accession ID: Q3I8J3), and A33R (Accession ID: Q3I8L8) proteins of the monkeypox virus were retrieved from UniProt, an online database (accessed on August 5, 2022). First, the aforementioned proteins were submitted to the VaxiJen server (http://www.ddg-pharmfac.net/vaxijen/VaxiJen/VaxiJen.html) to check their antigenicity (20). The overall steps involved in the present study from epitope prediction to vaccine construction and validation are shown in **Figure 1**. For the mentioned purpose, the NetCTL 1.2 (http://www.cbs.dtu.dk/services/NetCTL/) online server was used to predict the CTL epitopes sorted based on the combined score and A1 supertype was selected for the NetCTL epitope prediction (21). However, the IEDB online server (http://tools.iedb.org/mhcii/) was used for the prediction of HTL epitopes specific for human MHC molecules such as HLA-DRB1*03:01, HLA-DRB1*03:01, HLA-DRB1*15:01, HLA-DRB3*01:01, HLA-DRB3*02:02, HLA-DRB4*01:01 and HLA-DRB5*01:01 (22). The HTL epitopes were selected for vaccine construction based on the lowest percentile ranking. The lower percentile ranking depicts higher binding affinity of epitopes. Finally, the ABCPred server (http://crdd.osdd.net/raghava/abcpred/) was used to predict the B-cell epitopes that are important for the generation of protective human antibodies (23). The best epitopes were selected based on high scores, and the cutoff value was set to 0.8.

### Construction and characterization of the multiepitope subunit vaccine

After the prediction of immunogenic epitopes for the L1R, B5R, and A33R proteins, we used different linkers to combine the predicted epitopes and finalized the design of the MESVs. The B-cell, HTL and CTL epitopes were combined by using KK, GPGPG and AAY linkers, respectively. These linkers are immunogenic in nature and help the constructed vaccines boost immune responses. Second, these linkers prevent the folding of epitopes, keeping them separated from each other. Human beta (β)-defensin 2 has an important role in innate immunity and generates highly specific immune responses (24); therefore, to enhance the constancy and immunogenic response of the constructed proteins, we attached human β-defensin 2 to the N-terminal end of the vaccine sequence (25). Finally, the constructed vaccines for L1R, B5R, A33R, and a proteome-wide construct were used for immunogenicity and allergenicity validation using VaxiJen and Algpred (https://webs.iiitd.edu.in/raghava/algpred/submission.html), respectively. We utilized ProtParam tools (https://web.expasy.org/protparam/) to check the physiochemical properties of the designed proteins, such as half-life, theoretical PI, molecular weight, and grand average of hydropathy (GRAVY) (26).

### Prediction and validation of the 3D structure of the multiple-epitope vaccine

After verification of the immunogenicity and nonallergenicity of the constructed vaccines, the protein sequences of the designed vaccines were submitted to the Robetta server (https://robetta.bakerlab.org/) for 3D structural modeling. The Robetta server uses Continuous Automated Model EvaluatiOn (CAMEO) and has been recognized as the most precise and consistent server since 2014 (27). However, the Galaxy Refine sever was used to refine the protein quality (28). Afterward, to check the quality of the predicted vaccine model, we submitted the 3D structure of the constructed vaccines (L1R, B5R, A33R, and a proteome-wide construct) to the online validation tools ProSa-Web (https://prosa.services.came.sbg.ac.at/prosa.php) (29) and PROCHECK (https://servicesn.mbi.ucla.edu/PROCHECK/). The protein structure was analyzed by using the aforementioned servers based on the quality score.

### MD simulations of the modeled vaccine structure

MD simulations were performed using the AMBER 20 package to evaluate the protein folding of the constructed vaccine (30). Proper folding of a protein is necessary to carry out normal biological functions, such as cell-to-cell linkages, and trigger immunogenic responses (31). Before running the 50-ns simulation, the system was solvated using the FF19SB force field and the TIP3 box (12.0 A^⁰^) and then neutralized by using Na+ counter ions. Afterward, a two-step minimization (3000 and 6000 steps) was carried out to remove bad clashes, followed by heating (upto 300K) and equilibrations (1 atm pressure). Finally, the root-mean square deviation (RMSD) and root-mean square fluctuation (RMSF) were calculated by using the CPPTRAJ and PTRAJ packages respectively, which are embedded in AMBER 20, to check the stability, flexibility, and folding of constructed proteins (32).

### Molecular docking and dissociation constant (KD) analysis

Binding of the vaccine to the host immune cell receptor is necessary for the induction of human immune responses. Previously, the TLR-2 was recommended for the double-stranded DNA viruses belongs to the family Poxviridae so we selected the TLR-2 human receptor for our study (33). Therefore, to check the binding of the constructed vaccines (L1R, B5R, A33R, and a proteome-wide construct) with human TLR-2, we utilized the HDOCK sever (http://hdock.phys.hust.edu.cn/) (34). The HDOCK server supports the file as an amino acid sequence and then uses a template-based hybrid algorithm to check the interaction between proteins and protein−DNA/RNA, distinguishing this server from other available servers. To perform docking, first, the 3D structure of TLR-2 (PDB ID: 6nig) was retrieved from the UniProt online database (https://www.uniprot.org/) and submitted to the HDOCK server along with the designed vaccine proteins. To further verify the strength of vaccine-TLR complexes, we performed dissociation constant (KD) analysis. Herein, we used the online server PRODIGY (https://wenmr.science.uu.nl/) to calculate the KD value (35). To check whether our constructed vaccine can induce the human immune system through multiple TLRs, we also used TLR-3 for molecular docking with the constructed vaccines and followed the same method as for TLR-2.

### *In silico* cloning and codon optimizations

A computer-based genetic method called *in silico* codon optimization is primarily used to optimize particular amino acid sequences and achieve the highest expression of proteins. Hence, to clone the designed vaccine in the *Escherichia coli* (*E. coli*) K12 expression system, we used the Java codon adaptation tool (JCat) (http://www.jcat.de/) to convert the amino acid sequence of vaccines into a DNA sequence (36). The GC content and codon adaptation index (CAI) values generated by the JCat server represent the level of expression in the *E. coli* expression system. Finally, SnapGene software was used to clone the constructed vaccines in the pET-28a (+) expression vector.

### Immune simulation

To check the response of the human immune system against the constructed L1R, B5R, A33R, and proteome-wide vaccines, we used the online immune simulation server called C-ImmSim (https://kraken.iac.rm.cnr.it/C-IMMSIM/index.php?page=1) (37). The aforementioned server characterized the constructed vaccines in terms of their antigenicity and ability to trigger immune responses against the injected vaccines. This server estimates the level of immune cells such as helper T-cell 1 (Th1) and helper T-cell 2 (Th2). Moreover, among other immunological reactions, the server also determines the level of interferon, cytokines, and antibodies generated against administered vaccines.

## Results and discussion

Monkeypox cases have been reported in multiple countries, such as the US, the UK, Singapore and Israel during 2018-2021 (7), and monkeypox has subsequently spread to more than 50 countries since May 2022. Currently, no specific treatments are being devised to treat monkeypox. However, antivirals, VIG, and smallpox vaccines (Dryvax) are useful to control monkeypox outbreaks (2). However, the Dryvax has adverse side effects such as acquired or congenital defects in the immune system of both the vaccinee and others who are in close contact with the vaccinee. Additionally, the live virus vaccination is contagious and can be shared by the vaccinee with those in their immediate circle, including youngsters and those with compromised immune systems (38, 39). Due to the current pandemic situation of monkeypox, the development of vaccines against monkeypox virus is urgently needed. The immunoinformatic approach has several advantages over traditional vaccine development. Therefore, in the current study, we used an immunoinformatic approach to target the L1R, B5R, and A33R proteins of the monkeypox virus to screen for immunogenic and non allergenic CTL, HTL, and B-cell epitopes to construct MESVs against monkeypox virus.

### Sequence retrieval and characterization

To neutralize invading pathogens and clear them from the human body, acquired immune responses are very important. After the initial attack of the infectious agent, acquired immunity in humans produces memory cells that recognize the pathogens on subsequent attacks. These memory cells provide the basis for the development of a vaccine against specific pathogens (40, 41). Therefore, in the present study, we used immunoinformatic approaches to develop a MESVs to boost acquired immunity against the monkeypox virus. As previously verified, plasmid DNA that encodes the monkeypox ortholog L1R, B5R, and A33R proteins induced immunity in rhesus macaques (15). Therefore, to develop a highly immunogenic and nonallergenic MESVs, we retrieved the protein sequences of L1R, B5R, and A33R from the UniProt database. Then, the protein sequences were screened for antigenicity and allergenicity. The default threshold is 0.4, and the antigenicity scores for L1R, B5R and A33R were 0.737, 0.425 and 0.447, respectively. Furthermore, all three proteins were found to be nonallergenic.

### Immunogenic epitope predictions

In acquired immunity, the MHC-II proteins that are present in all nucleated cells support active immunity by presenting epitopes of invading pathogens to CTLs (42). Due to the importance of CTLs in the clearance of the invading pathogens, we submitted the protein sequences of L1R, B5R, and A33R for prediction of highly immunogenic CTL epitopes. Among the predicted CTL epitopes, 4 epitopes were selected for each of the L1R (TMADDDSKY, SDDSSSDTY, AATTADVRY, and AVGHCYSSY), B5R (LSSINIKEY, CTLLHRCIY, YILDTVNIY, and ILDWSIYLF) and A33R (MLFYMDLSY, KVNNNYNNY, IVTKVNNNY, and YMDLSYHGV) proteins based on high MHC binding affinity and prediction score (**Table 1**). Moreover, the CTL epitopes were selected for the proteome-wide vaccine from the aforementioned predicted epitopes (two epitopes from each of L1R, B5R, and A33R) based on high MHC binding affinity and prediction scores (**Table 1**).

**Table 1:** List of CTL epitopes predicted for the L1R, B5R, and A33R proteins of monkeypox virus.

Residue no.	Peptide sequence	MHC binding affinity	Rescaled binding affinity	C-terminal cleavage affinity	Transport affinity	Prediction score	MHC-I binding
**L1R**							
1	TMADDDSKY	0.3527	1.4976	0.9768	2.990	1.793	YES
16	SDDSSSDTY	0.2113	0.8970	0.9505	2.6480	1.1720	YES
70	AATTADVRY	0.1808	0.7675	0.9721	3.1390	1.0703	YES
30	AVGHCYSSY	0.1607	0.6821	0.9626	3.1450	0.9838	YES
**B5R**							
174	LSSINIKEY	0.4865	2.0657	0.7181	2.9350	2.3201	YES
11	CTLLHRCIY	0.2804	1.1905	0.5104	2.8500	1.4096	YES
147	YILDTVNIY	0.2639	1.1203	0.8592	3.1050	1.4045	YES
8	ILDWSIYLF	0.1901	0.8071	0.7902	2.2340	1.0373	YES
**A33R**							
75	MLFYMDLSY	0.2906	1.2337	0.9678	3.1390	1.5358	YES
111	KVNNNYNNY	0.2570	1.0913	0.8559	3.0860	1.3740	YES
108	IVTKVNNNY	0.2470	1.0486	0.9299	3.0600	1.3411	YES
78	YMDLSYHGV	0.2674	1.1353	0.8065	0.1350	1.2631	YES
**Proteome**							
1	TMADDDSKY	0.3527	1.4976	0.9768	2.990	1.793	YES
16	SDDSSSDTY	0.2113	0.8970	0.9505	2.6480	1.1720	YES
174	LSSINIKEY	0.4865	2.0657	0.7181	2.9350	2.3201	YES
11	CTLLHRCIY	0.2804	1.1905	0.5104	2.8500	1.4096	YES
75	MLFYMDLSY	0.2906	1.2337	0.9678	3.1390	1.5358	YES
111	KVNNNYNNY	0.2570	1.0913	0.8559	3.0860	1.3740	YES

On the other hand, helper T-cells are also important in generating acquired immunity against invading pathogens including monkeypox virus. Helper-T cells are involved in activating both the B-cell and cytotoxic T-cell pathways. After the entry of monkeypox virus into the human body, macrophages engulf the this virus and present the epitopes to helper T-cells to activate the B-cells to produce neutralizing antibodies. Due to the indispensable role of HTLs in boosting immune responses, we submitted the proteins L1R, B5R, and A33R for prediction of the HTL epitopes that trigger immune responses. Among the predicted epitopes, we selected only four epitopes with the lowest percentile rank for each of the L1R (ADVRYDRRDASNVMD, AATTADVRYDRRDAS, DKKNKVVTDGRVKNK, and KNKVVTDGRVKNKGY), B5R (NRGSFIHNLGLSSIN, FLSYILDTVNIYISI, PSLKIMIPSMIAITK, and LHIYFIDAANTNIMI) and A33R (YNCYNYDDTFFDDDD, GKVTINDLKMMLFYM, VLGKVTINDLKMMLF, and SGGGIYHDDLVVLGK) proteins. Similarly, two epitopes with the lowest percentile score from each of the above proteins were selected for the construction of a proteome-wide vaccine (**Table 2**).

**Table 2:** List of Th-cell epitopes predicted for the L1R, B5R, and A33R proteins of monkeypox virus .

S. no.		Allele	Start	End	Peptide sequence	Method	Percentile rank	
**L1R**								
1	HLA-DRB1*03:01		74	88	ADVRYDRRDASNVMD	Consensus (comb.lib./smm/nn)		0.34
2	//		70	84	AATTADVRYDRRDAS	//		0.34
3	//		45	59	DKKNKVVTDGRVKNK	//		1.9
4	//		47	61	KNKVVTDGRVKNKGY	//		1.9
**B5R**								
1	HLA-DRB3*02:02		99	113	NRGSFIHNLGLSSIN	NetMHCIIpan		0.01
2	HLA-DRB3*01:01		144	158	FLSYILDTVNIYISI	Consensus (smm/nn/sturniolo)		0.02
3	//		400	414	PSLKIMIPSMIAITK	//		0.06
4	//		73	87	LHIYFIDAANTNIMI	//		0.19
**A33R**								
1	HLA-DRB3*01:01		128	142	YNCYNYDDTFFDDDD	//		0.52
2	HLA-DRB1*03:01		65	79	GKVTINDLKMMLFYM	//		0.86
3	//		63	77	VLGKVTINDLKMMLF	//		0.91
4	HLA-DRB3*01:01		52	66	SGGGIYHDDLVVLGK	//		2.40
**Proteome**								
1	HLA-DRB1*03:01		74	88	ADVRYDRRDASNVMD	//		0.34
2	//		70	84	AATTADVRYDRRDAS	//		0.34
3	HLA-DRB3*02:02		99	113	NRGSFIHNLGLSSIN	NetMHCIIpan		0.01
4	HLA-DRB3*01:01		144	158	FLSYILDTVNIYISI	Consensus (smm/nn/sturniolo)		0.02
5	//		128	142	YNCYNYDDTFFDDDD	//		0.52
6	HLA-DRB1*03:01		65	79	GKVTINDLKMMLFYM	//		0.86

Differentiated B-cell are the sole cells responsible for producing antibodies in our body and are considered key players in the adaptive and innate immune responses (43). Furthermore, the design of B-cell epitopes is very important for the generation of immune responses because the antibodies that are produced by B cells induce some protective mechanisms, such as agglutination, neutralization, complement system activation, and cell-mediated cytotoxicity (44). Therefore, all the selected proteins of the monkeypox virus were submitted for the prediction of B-cell epitopes. Among the predicted epitopes, we selected epitopes with a score greater than 0.8 and used them for designing the final vaccine against the monkeypox virus (**Table 3**). Finally, we selected one epitope from each protein with a high score for proteome-wide vaccine construction.

**Table 3:** List of B-cell epitopes predicted by the ABCpred server.

S. no.	Position	Epitope	Score	
**L1R**				
1	10	DDSKYASDDSSSDTYDSKNK		0.87
2	37	SYRTKTDSDKKNKVVTDGRV		0.86
**B5R**				
1	60	PNLFHILEYGENILHIYFID		0.82
2	441	AITKHKQHNADLLKMCIKYT		0.82
**A33R**				
1	53	GGGIYHDDLVVLGKVTINDL		0.91
2	37	FVFYKPKHSTVVKYLSGGGI		0.85
**Proteome**				
1	10	DDSKYASDDSSSDTYDSKNK		0.87
2	60	PNLFHILEYGENILHIYFID		0.82
3	53	GGGIYHDDLVVLGKVTINDL		0.91

### Construction of the MESVs and physiochemical parameter analysis

**Figure 2:**
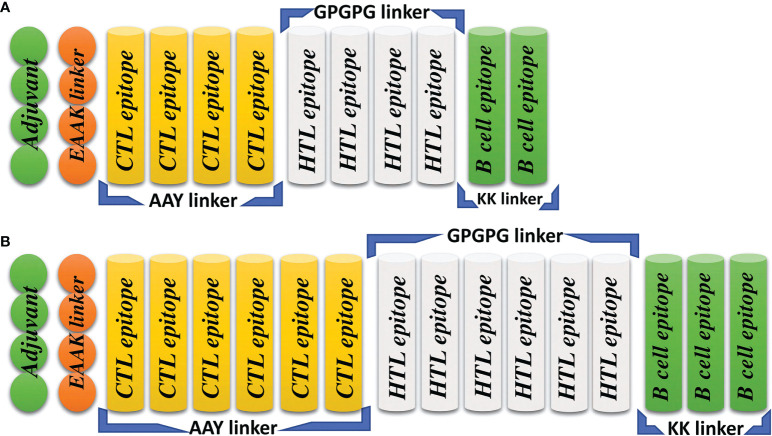
Topographical representation of the final vaccine constructs. **(A)** The final vaccine construct of L1R, B5R and A33R. **(B)** The final proteome-wide vaccine construct.

The computer-aided design of vaccines is a more appropriate replacement for traditional vaccine development, as it is cost-effective, results in the combination of highly immunogenic epitopes from different regions of the protein, is less time consuming and results in the production of no allergenic responses (45). To construct the final MESVs, we combined all the predicted immunogenic epitopes for L1R, B5R, A33R and the proteome-wide construct by using different linkers. The AAY, GPGPG, and KK linkers were used to combine CTL, HTL, and B-cell epitopes, respectively. These linkers are immunogenic in nature and help the constructed vaccines boost immune responses like the AAY liker enhances the epitope presentation, the GPGPG linker stimulates the HTL responses and the immunogenicity of antibody epitopes while the KK linker enhances the constructed vaccines immunogenicity. Second, these linkers prevent the folding of epitopes, keeping them separated from each other (42, 46). Finally, the EAAK linker was used to connect the adjuvant to the N-terminal end of the constructed vaccine, which further enhances the efficiency of the vaccine in triggering immune responses. Human β-defensin 2 has an important function in innate immunity and can generate antigen-specific immune responses (24); therefore, to increase the stability and immunogenic response of the constructed vaccine, we attached human β-defensin 2 as an adjuvant (**Figure 2**) (25). Finally, to provide a highly immunogenic vaccine with no allergenic responses, the constructed vaccines were subjected to allergenicity and antigenicity checks. The constructed vaccines were found to be nonallergenic, with an antigenicity score higher than the threshold value (0.4).

Then, the physiochemical properties of the constructed vaccines were calculated, such as half-life, theoretical PI, molecular weight, aliphatic index, instability index, and GRAVY. The calculated molecular weights for L1R, B5R, A33R, and the proteome-wide construct were 31.9, 32.4, 32.1, and 41.7 kDa, respectively, while the half-life of all constructed vaccines was greater than 10 hours in *E. coli.* This means that the constructed vaccine can be easily expressed and purified, as the molecular weights are within the normal range. An instability index value of less than 40 indicates that a protein is stable. The instability index values were less than 40, which means that the constructed vaccines are stable (**Table 4**). The theoretical PI value, aliphatic index, and GRAVY represent the acidity, thermostability and hydrophobicity of the constructed vaccines, respectively (26). The values of the physiochemical properties for L1R, B5R, A33R, and the proteome-wide construct are shown (**Table 4**).

**Table 4:** Designed vaccine physiochemical properties.

Vaccine construct	Molecular weight (kDa)	Theoretical PI	Half-life in *E. coli*	Instability index	Aliphatic index	GRAVY
L1R	31.9	5.39	>10 hours	29.27	65.3	-0.584
B5R	32.4	5.69	>10 hours	31.59	108.38	0.263
A33R	32.1	4.98	>10 hours	24.46	89.50	0.023
Proteome wide	41.7	4.60	>10 hours	37.13	80.34	-0.253

### 3D structure prediction and validation

**Figure 3:**
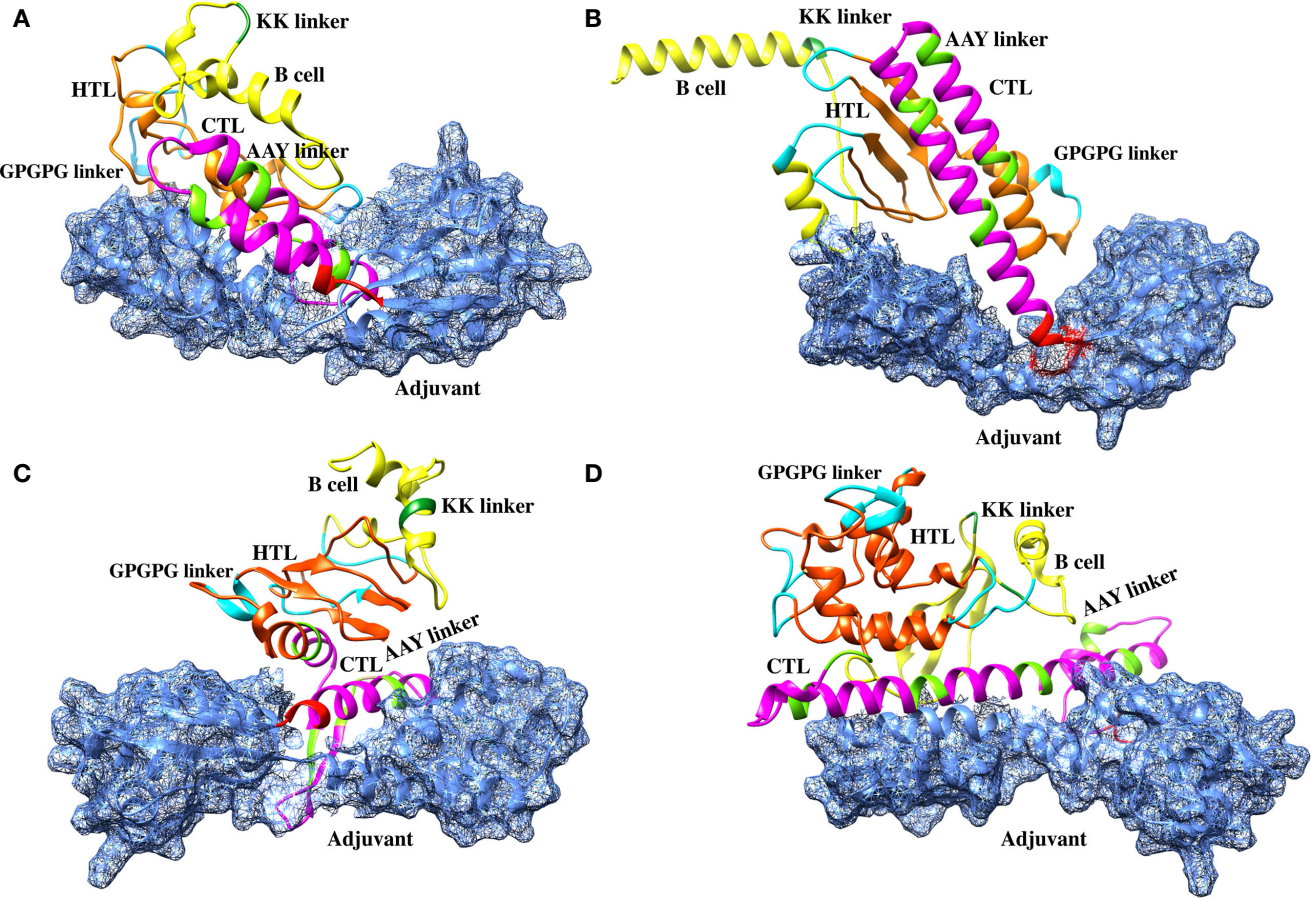
Robetta-generated 3D structures for the final vaccine constructs of the monkeypox virus. **(A)** The 3D structure of the L1R final vaccine construct generated by Robetta. **(B)** The 3D structure of the B5R final vaccine construct generated by Robetta. **(C)** The 3D structure of the A33R final vaccine construct generated by Robetta. **(D)** The 3D structure of the proteome-wide final vaccine construct generated by Robetta.

The sequences of the predicted vaccines L1R, B5R, A33R, and the proteome-wide construct were submitted to the Robetta server for 3D structure modeling. The Robetta server uses CAMEO and has been recognized as the most precise and consistent server since 2014 (27). The Robetta server generated five models for each of the constructed vaccines, and we selected the best model for each vaccine based on quality analysis (**Figure 3**).

**Figure 4:**
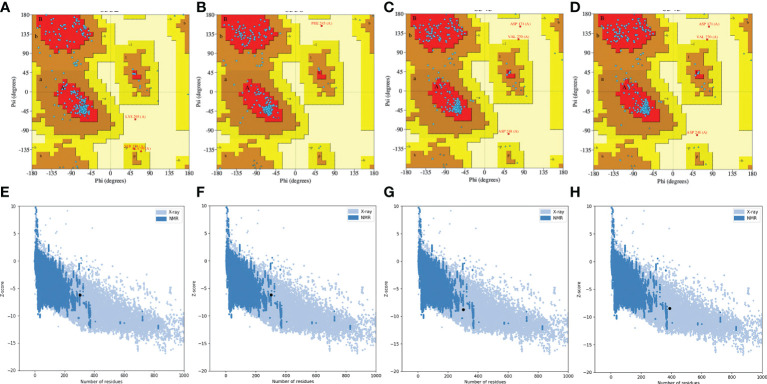
Quality analysis of Robetta-generated models by Ramachandran plots and ProSA-web. **(A–D)** Structural validation of the L1R, B5R, A33R, and proteome-wide vaccines by Ramachandran plots. **(E–H)** Structural validation of the L1R, B5R, A33R, and proteome-wide vaccines by ProSA-web. Uppercase and lowercase: This is the international farmate for this sever. ProSA-web (protein structure analysis-web).

To select the best model for the designed vaccines, we performed ProSA-web and Ramachandran plot analyses. First, we submitted the models for Ramachandran analysis and selected the model with the highest ratio of amino acids present in the favored region and the lowest ratio in the disallowed region. The percentage of residues in the favored region was 87.5%, 92.4%, 88.5%, and 85.4% for L1R, B5R, A33R, and the proteome-wide construct, respectively; however, the percentage of residues present in the outlier region was 0.4%, 0.4%, 1.2%, and 0.9%, respectively. Subsequently, to confirm the quality and errors in the predicted structures, we submitted the models for ProSA-web analysis. The predicted quality Z score was -6.19, -6.17, -8.77, and -8.42 for L1R, B5R, A33R, and the proteome-wide construct, respectively (**Figure 4**). The above data indicated that the quality of the selected structures is good, as the Z score lies in the normal range. Furthermore, to check the proper folding of the model structures, we subjected the selected models to molecular dynamics simulation analysis.

### Molecular dynamics simulations

**Figure 5:**
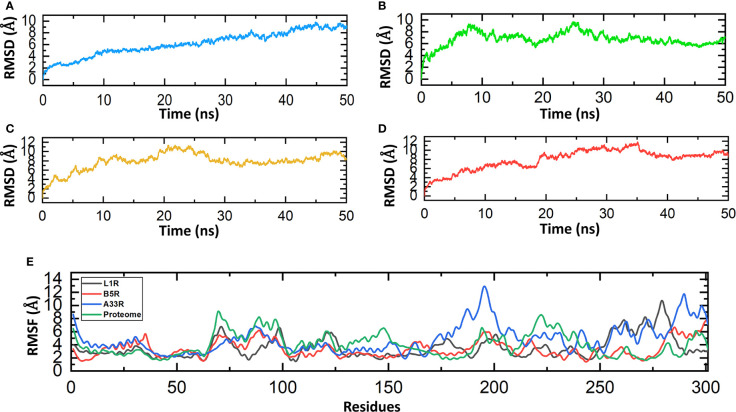
Molecular dynamics simulations of the constructed vaccines. **(A)** RMSD value for the L1R vaccine construct. **(B)** RMSD value for the B5R vaccine construct. **(C)** RMSD value for the A33R vaccine construct. **(D)** RMSD value for the proteome-wide vaccine construct. **(E)** RMSF values for the L1R, B5R, A33R and proteome-wide vaccine constructs.

The stability investigation of the constructed vaccines by MD simulations revealed the stable behavior of the B5R, A33R and proteome-wide vaccines; however, a steady fluctuation was observed in the L1R vaccine until 50 ns. The B5R protein gained stability at 9 ns and remained stable until 50 ns. However, the protein A33R and proteome-wide gained stability at 20 ns and 30 ns and remained stable until 50 ns. The above data confirmed the stable folding and sustained behavior during the course of simulations (**Figures 5A–D**). The residual fluctuations of the constructed vaccines were also evaluated by calculating the RMSF. The residual fluctuations were found to be minimal; however, little fluctuation was found between 175-200 and 250-300. The residues present at the ends of proteins show more fluctuations, which might be due to the flexible nature of the N-terminal and C-terminal ends (**Figure 5E**).

### Molecular docking of constructed vaccines with human TLR-2

**Figure 6:**
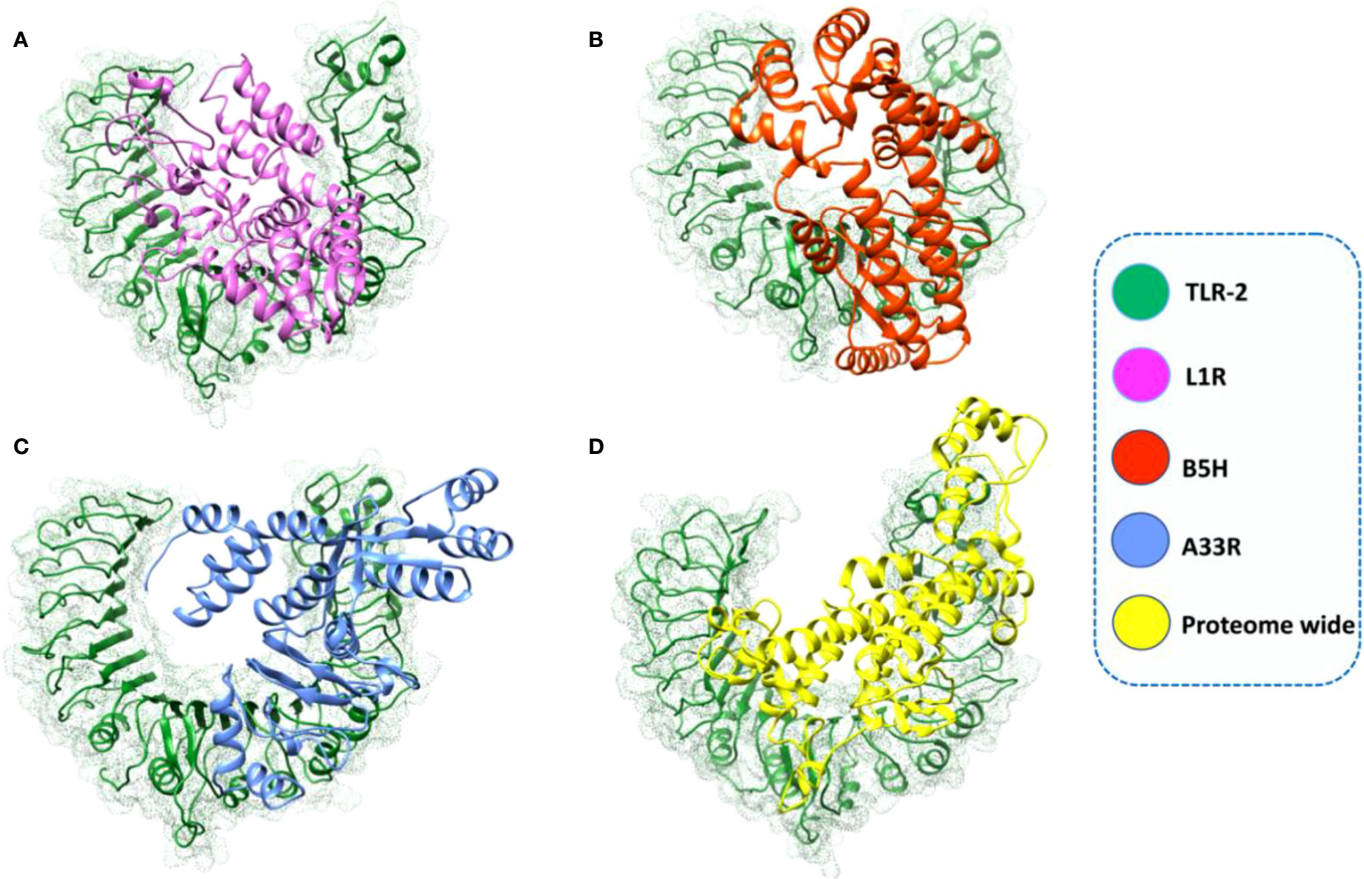
Complexes of the L1R, B5R, A33R, and proteome-wide vaccine constructs with human TLR-2. The designed vaccines and TLR-2 are shown in different colors. **(A)** L1R-TLR-2 complex; **(B)** B5R-TLR-2 complex; **(C)** A33R-TLR-2 complex; **(D)** proteome-wide construct-TLR-2 complex.

Monkeypox virus after entering the host infect the dendritic cells (DCs), macrophages and also inhibit the activation of host innate immune system (47, 48). TLRs are important regulators of inflammatory pathways that play indispensable roles in mediating immune responses to pathogens. TLRs recognize pathogen-associated molecular patterns (PAMPs), leading to changes in gene expression and triggering intracellular signaling pathways. The innate immune system of the host detects pathogens and responds accordingly *via* recognition by TLRs (49). TLRs are important in recognizing the various components of monkeypox viruses, such as nucleic acids and envelope glycoproteins, leading to a series of cascades, including the production of IFN-I, inflammatory cytokines and chemokines (50). Moreover, adaptive immune responses are also activated when TLRs induce the maturation of dendritic cells (DCs) (51). Therefore, to check the binding affinity of the constructed vaccines with human TLR-2, we used the HDOCK server. The binding scores predicted by HDOCK for the L1R, B5R, A33R, and proteome-wide vaccines were -266 kcal/mol, -288 kcal/mol, -264 kcal/mol, and -250 kcal/mol, respectively, which shows robust binding of all constructed vaccines with human TLR-2 (**Figure 6**). The above data were further verified by interface analysis using the PDBsum server. The binding interface analysis of the L1R-TLR-2 complex revealed 10 hydrogen bonds, 2 salt bridges and 213 non-bonded contacts. However, the B5R-TLR-2 complex formed 1 hydrogen bond, 3 salt bridges and 167 non-bonded contacts. The bonding network of the A33R-TLR-2 complex formed 3 hydrogen bonds, 2 salt bridges and 172 non-bonded contacts. Similarly, the bonding network of the proteome-wide construct-TLR-2 complex formed 8 hydrogen bonds, 5 salt bridges, and 216 non-bonded contacts. To further verify the strength of a vaccine-TLR complex, we performed dissociation constant analysis. The K_D_ values for L1R-TLR-2, B5R-TLR-2, A33R-TLR-2 and the proteome-wide construct-TLR-2 complex were 4.1E^-10^, 5.1E^-09^, 3.4E^-08^, and 1.5E^-06^, respectively. The K_D_ estimation revealed that the L1R-TLR-2 (4.1E^-10^) complex had a stronger binding affinity than all the other complexes. Furthermore, to check whether our constructed vaccine can induce the human immune responses through multiple TLRs, we docked the constructed vaccines with human TLR-3. The binding scores of L1R, B5R, A33R, and proteome-wide vaccines with human TLR-3 were -241 kcal/mol, -291 kcal/mol, -255 kcal/mol, and -261 kcal/mol, respectively. The analysis of binding interface revealed that the L1R-TLR-3 complex contains 4 salt bridges, 6 hydrogen bonds and 130 non-bonded contacts, while the B5R-TLR-3 complex contains 6 salt bridges, 7 hydrogen bonds and 343 non-bonded contacts. Similarly, the A33R-TLR-3 complex contains 1 hydrogen bond and 118 non-bonded contacts however, the proteome-TLR-3 complex contains 5 salt bridge, 12 hydrogen bonds and 244 non-bonded contacts. The dissociation constant analysis was performed to check the strength of the aforementioned complexes. The KD values for the LIR-TLR-3, B5R-TLR-3, A33R-TLR-3 and proteome-TLR-3 complexes were 1.6E^-09^, 2.2E^-06^, 3.8E^-07^, and 4.5E^-12^, respectively. In summary, the bonding network and dissociation constant analysis revealed that our constructed vaccines can induce the human immune system through multiple TLRs however, the binding affinity of constructed vaccines for TLR-2 is stronger as compared to the TLR-3 except proteome wide vaccine. Secondly, in case of TLR-3 the bonding target of constructed vaccines in not uniform. Therefore, our study verifies the previous recommendation of TLR-2 for the double stranded DNA envelope viruses belongs to the family Poxviridae (33).

### *In silico* cloning of MESVs in the pET-28a (+) expression vector

**Figure 7:**
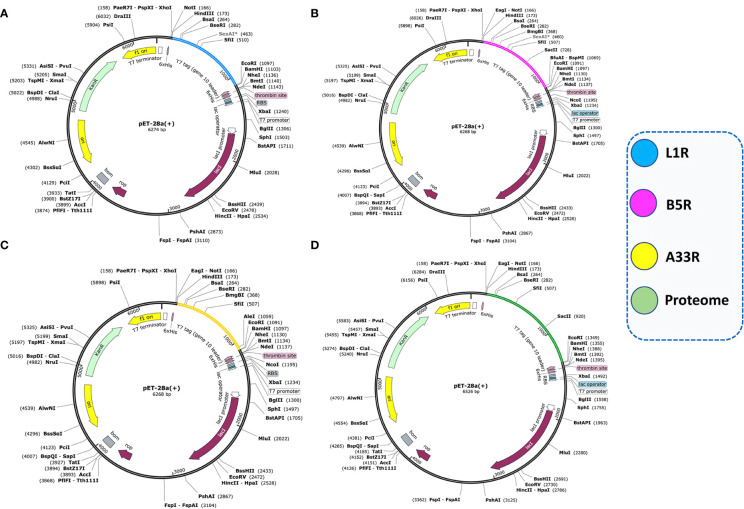
Cloning of optimized vaccine sequences in the pET28a (+) expression system. **(A)** Cloning of the L1R vaccine sequence in the pET28a (+) vector. **(B)** Cloning of the B5R vaccine sequence in the pET28a (+) vector. **(C)** Cloning of the A33R vaccine sequence in the pET28a (+) vector. **(D)** Cloning of the proteome-wide vaccine sequence in the pET28a (+) vector.

Codon optimization of the constructed vaccines (L1R, B5R, A33R, and the proteome-wide construct) was carried out for maximum expression in the *E. coli* K12 strain expression system by using the JCat tool. The JCat tool calculates some parameters, such as the CAI value and GC content (36). The generated CAI values for L1R, B5R, A33R and the proteome-wide vaccine construct were 0.96, 0.95, 0.96, and 0.95, while the GC contents were 69.9%, 65%, 65.7% and 66.1%, respectively. The estimated CAI values for the above constructed vaccines lie in the ideal range for high expression; however, the ideal range of GC content is from 30%-70%. Herein, the above data confirmed the high expression of the constructed vaccines in the *E. coli* expression system. Then, the optimized sequences were cloned into the pET-28a (+) expression vector using the *Xho1* and *EcoR1* restriction sites (**Figure 7**).

### Immune response simulation analysis

**Figure 8:**
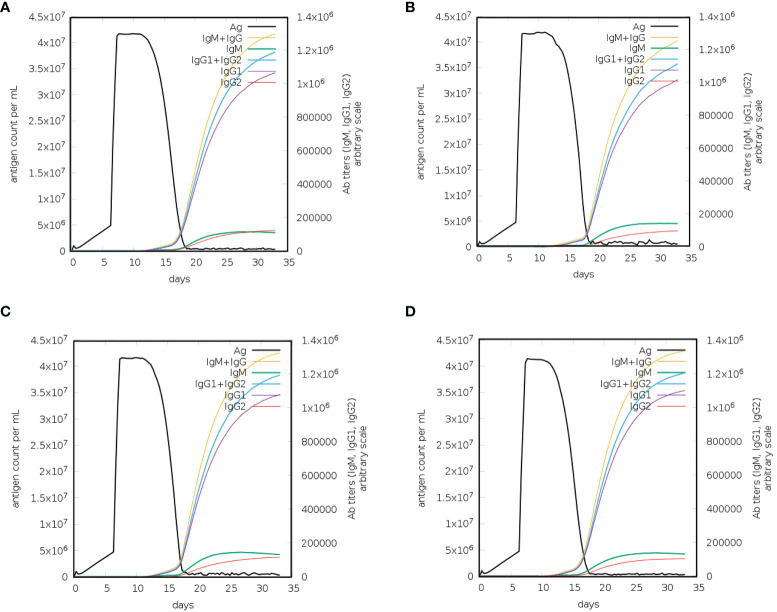
Immune response simulation for a constructed vaccine against monkeypox virus. **(A)** Immune responses to the L1R vaccine, **(B)** immune responses to the B5R vaccine, **(C)** immune responses to the A33R vaccine, and **(D)** immune responses to the proteome-wide vaccine.

Vaccination elicits immune responses in the body without causing the disease. Different vaccines stimulate immune responses differently (52). Protein antigens together with adjuvants trigger local innate immune responses, i.e., proinflammatory cytokine production by macrophages. Dendritic cells or macrophages take up the antigen, and these cells then present the antigen on their surface *via* MHC molecules. T-cells recognize the MHC/antigen complex *via* their T-cell receptors, leading to the production of memory T-cells (adaptive immunity). On the other hand, antigens comprising polysaccharides induce antibody responses without T-cell involvement. Polysaccharides bind to mature B-cells, leading to the production of antibody-producing B cells. Such immunological responses do not have memory (53). Immune response simulation analysis was performed to check the immune boosting efficiency of the constructed vaccines. The immune simulation monitors the response of the immune system in terms of antibody production after the injection of constructed vaccines. After the injection of each vaccine construct, the response of the immune system and antibody production were extremely high (**Figure 7**). After injection of each construct, the antigen titer remained low until the 5^th^ day, and then the titer increased until the 13^th^ day; however, the increase in the antibody titer started on the 15^th^ day. On the 17^th^ day, the total neutralization of antigens was recorded with the induction of the production of other immune system factors. The combined IgM and IgG titer reached 4× 10^7^ in the case of L1R and B5R, while the combined IgM and IgG titer reached 4.3× 10^7^ after injection of A33R and the proteome-wide vaccine (**Figure 7**). High combined IgG1+IgG2 and IgG1 titers were also observed after injection of the L1R, B5R, A33R, and proteome-wide vaccines. The level of cytokines and interleukins were also analyzed after the injection of constructed vaccines (**Supplementary figure 2**). At 5^th^ day of injection the level of IFN-γ and IL-2 increased slowly and reached to maximum at 18^th^ day. The levels of IFN-γ and IL-2 were higher in case of A33R vaccine as compared to the others. The levels of aforementioned immune factors were significantly higher, which shows the vigorous and steady immune triggering response upon injection. The above data showed that the constructed vaccines can robustly induce immune responses against the invading monkeypox virus (**Figure 8**).

## Conclusions

Computationally designed vaccines are thermodynamically stable, effective, specific and low cost compared to traditional vaccine development. In the current study, we used immunoinformatic approaches to design highly immunogenic, thermostable and nonallergenic MESVs against the monkeypox virus. We used L1R, B5R, and A33R proteins of monkeypox virus to develop highly antigenic and nonallergenic CTL, HTL and B-cell epitopes by using bioinformatics tools. Various bioinformatics tools and approaches confirmed that the 3D structure of each constructed vaccine had a proper folding, a stronger binding with human TLR-2 and high expression of constructed proteins in the *E. coli* expression system. Moreover, the injected vaccines robustly activated the immune system, with high titers of IgG and IgM antibodies. In conclusion, the present study provided dynamic and effective vaccines that are composed of highly antigenic and non-allergenic peptides against the monkeypox virus, demanding further experimental trials.

## Data availability statement

The original contributions presented in the study are included in the article/**Supplementary Material**. Further inquiries can be directed to the corresponding author.

## Author contributions

ZX presented the concept and edited the article. MS presented the concept, analyzed the data, and wrote the manuscript. FR, SA, and HS designed the figures and edited the Manuscript. SL and LX review the manuscript and contributed to the data analysis. All authors contributed to the article and approved the submitted version.

## Funding

This work was supported by the Guangxi BaGui Scholars Program Foundation (2019A50).

## Conflict of interest

The authors declare that the research was conducted in the absence of any commercial or financial relationships that could be construed as a potential conflict of interest.

## Publisher’s note

All claims expressed in this article are solely those of the authors and do not necessarily represent those of their affiliated organizations, or those of the publisher, the editors and the reviewers. Any product that may be evaluated in this article, or claim that may be made by its manufacturer, is not guaranteed or endorsed by the publisher.

## Supplementary material

Supplementary Figure 1 Complexes of the L1R, B5R, A33R, and proteome-wide vaccine constructs with human TLR-3. The designed vaccines and TLR-3 are shown in different colors. **(A)** Showing L1R-TLR-3 complex; **(B)** Showing B5R-TLR-3 complex; **(C)** Showing A33R-TLR-3 complex; **(D)** Showing proteome-wide construct-TLR-3 complex.

Supplementary Figure 2 Response of human interleukins and cytokines upon injection of constructed vaccines. **(A)** Showing concentration of interlcukins after L1R vaccine injection. **(B)** Showing concentration of interlcukins after B5R vaccine injection. **(C)** Showing concentration of interlcukins after A33R vaccine injection. **(D)** Showing concentration of interlcukins after proteome wide vaccine injection.
