# Supplementary figures and images for: Immunoinformatic-based design of immune-boosting multiepitope subunit vaccines against monkeypox virus and validation through molecular dynamics and immune simulation
Source: Front Immunol. 2022 Oct 13;13:1042997. doi: 10.3389/fimmu.2022.1042997 (PMC9606240; doi:10.3389/fimmu.2022.1042997)

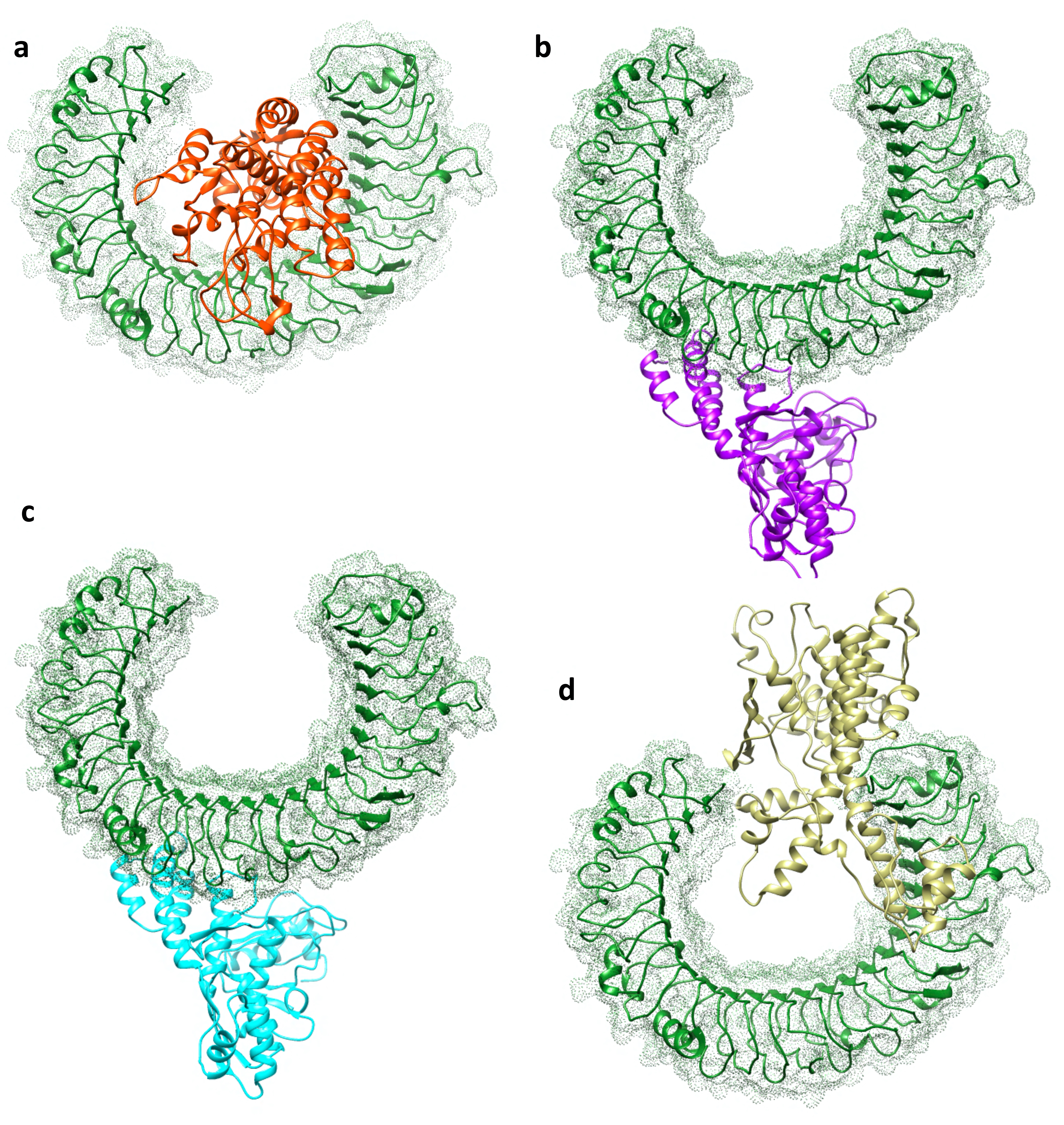

Supplement: Supplementary Figure 1 — Complexes of the L1R, B5R, A33R, and proteome-wide vaccine constructs with human TLR-3. The designed vaccines and TLR-3 are shown in different colors. (A) Showing L1R-TLR-3 complex; (B) Showing B5R-TLR-3 complex; (C) Showing A33R-TLR-3 complex; (D) Showing proteome-wide construct-TLR-3 complex. [file Image_1.jpg]

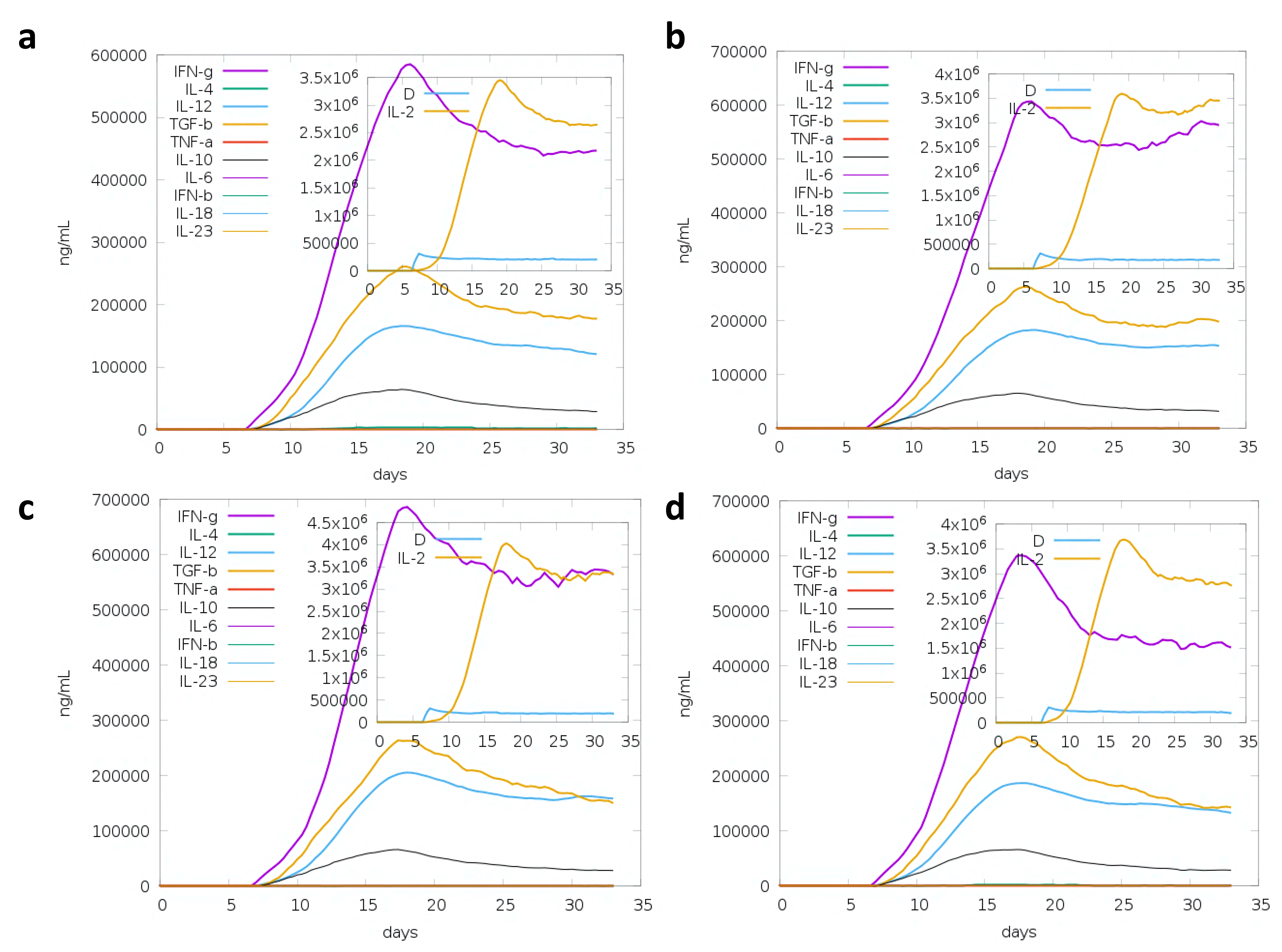

Supplement: Supplementary Figure 2 — Response of human interleukins and cytokines upon injection of constructed vaccines. (A) Showing concentration of interlcukins after L1R vaccine injection. (B) Showing concentration of interlcukins after B5R vaccine injection. (C) Showing concentration of interlcukins after A33R vaccine injection. (D) Showing concentration of interlcukins after proteome wide vaccine injection. [file Image_2.jpg]
